# Strategy Escalation: An emerging paradigm for safe clinical development of T cell gene therapies

**DOI:** 10.1186/1479-5876-8-55

**Published:** 2010-06-10

**Authors:** Richard Paul Junghans

**Affiliations:** 1 Departments of Surgery and Medicine, Boston University School of Medicine, Roger Williams Medical Center, Providence, RI 02908, USA

## Abstract

Gene therapy techniques are being applied to modify T cells with chimeric antigen receptors (CARs) for therapeutic ends. The versatility of this platform has spawned multiple options for their application with new permutations in strategies continually being invented, a testimony to the creative energies of many investigators. The field is rapidly expanding with immense potential for impact against diverse cancers. But this rapid expansion, like the Big Bang, comes with a somewhat chaotic evolution of its therapeutic universe that can also be dangerous, as seen by recently publicized deaths. Time-honored methods for new drug testing embodied in Dose Escalation that were suitable for traditional inert agents are now inadequate for these novel "living drugs". In the following, I propose an approach to escalating risk for patient exposures with these new immuno-gene therapy agents, termed Strategy Escalation, that accounts for the molecular and biological features of the modified cells and the methods of their administration. This proposal is offered not as a prescriptive but as a discussion framework that investigators may wish to consider in configuring their intended clinical applications.

## Introduction

Gene therapy techniques are being applied to modify T cells with chimeric antigen receptors (CARs) for therapeutic ends (designer T cells, T-bodies). At their simplest, CARs are an immunoglobulin binding domain fused to the zeta signaling chain of the T cell receptor ("IgTCR") that can redirect T cell killing against antibody-specified targets [1]. The versatility of this platform has spawned multiple options for their application. For the same target and CAR recognition domain, a diversity of signaling domains, co-expressed cytokines and anti-apoptotic genes may impact the survival and activity of the designer T cells, whereas other, adjunctive, procedures may support the stable engraftment of vast numbers of these effectors in vivo.

Time-honored methods of Phase I safety testing have relied on Dose Escalation of new drugs to protect patients while advancing therapeutic aims. However, these methods designed for short-acting inert agents are no longer sufficient with the advent of engineered cellular therapies that are "living drugs" with potential for lifelong exposures. Strategies applying different CARs and different means of their application may have different potentials for benefit, but which may also be paralleled in their potentials for harm. For these novel cellular agents, I propose a new concept to be added to the clinical trialist's lexicon: Strategy Escalation.

## Discussion

### Designer T cells and safety

The application of adoptive cellular therapies in any format may have generic consequences with constitutional symptoms from cytokines released or co-administered. For the most part, these are manageable in experienced hands and present no new challenges. What is new is that specificities can be engineered into T cells in analogous fashion to monoclonal antibodies that have been adapted to target selected tumor antigens. These antigens are typically normal cell constituents that are enriched in tumors. From a T cell perspective, CARs allow bypassing of thymic editing that prevents normal T cells from high avidity reactions against self-tumor, but that primarily protects from such reactions against self-tissue ("tolerance").

This bypassing of normal tolerance means that *some antigen targets may be unsafe for designer T cells*. This was recently shown in a designer T cell trial against G250, a prominent renal cell carcinoma antigen [2]. Antibody against G250 had been applied in humans without toxicity, but when this specificity was tested in designer T cell format, reaction occurred against low level G250 on biliary epithelium. This resulted in an intolerable hepatotoxicity in two of three patients with low infused doses in the range of 10^9^ cells (100-fold below typical Surgery Branch TIL doses [3]), necessitating dose reductions and, in one case, systemic steroids for T cell suppression. When steroids were removed, the patient had no resurgence of liver attack - but also no tumor response.

This key study illustrated that designer T cells carried the potential for serious toxicity. The safety of comparable Phase I interventions against other antigens (folate binding protein [4], Tag72 [5], CEA [6], CD171 [7] and GD2 [8]) indicate that *toxicity is a function of the target *- with no obvious means to predict which. The G250 toxicity also demonstrated that *safety of a target with antibody is no assurance of safety with designer T cells *[2]. This latter conclusion is not surprising given the indirect means of antibody toxicity [9] in comparison with the direct cytotoxic potency of T cells that also brings far greater sensitivity, killing with just a few antigen molecules per cell, far below immunohistochemical detection thresholds [10].

This G250 agent was expertly managed via a dose escalation plan in a Phase I setting; the system worked: no one died. Instead, it is the evolution of more complex Strategies that raise the special concerns of this essay.

### The Strategies

New Strategies evolved because several so-called 1^st ^generation IgTCR designer T cells (above) had been tested in the clinic without major tumor regressions. Two contributing problems were identified. Firstly, the infused designer T cells initially distributed widely through the blood and tissues, but then they quickly perished in the host that is already replete with T cells. Secondly, the few T cells that trafficked into tumor could initially exhibit killing, but they ultimately disappeared via a process of activation-induced cell death (AICD) or passed to a resting, inactive state.

These two problems prompted two corresponding *hypotheses* for improving tumor responses:

(1) *Responses could be improved*: if sufficient T cells were maintained systemically to sustain T cell percolation into tumor (although T cells survived for only a few days of tumor cell killing).

(2) *Responses could be improved*: if T cells were to activate and proliferate on antigen contact in tumor (although T cells in tumor were few in starting number).

To address hypothesis #1, Dudley, Rosenberg and colleagues [11] applied "conditioning" to create a "hematologic space" with high dose chemotherapy and/or whole body irradiation prior to T cell infusion in their TIL studies in melanoma. With the burst of IL7 and IL15 that accompanies the lymphopenic state [12], the infused T cells rode the recovery with a homeostatic expansion, i.e., independent of antigen stimulation. As such, low doses of infused T cells could expand 100-fold in vivo to become a stable, "engrafted" component of the lymphoid compartment, in some instances >50% of the cells that would be the equivalent of 5 × 10^11^ (0.5 kg!) tumor-specific T cells. This in turn led to dramatically improved tumor response rates with substantial numbers of durable remissions.

To address hypothesis #2, so called 2^nd ^generation "2-signal" CARs were created to improve their function [13]. To the basic TCRz signaling (Signal 1) of the IgTCR was added a co-stimulation Signal 2 via CD28 and/or other signaling domains, e.g., IgCD28TCRz. Signal 1 suffices for T cell killing, but Signal 1 + 2 engages the T cell proliferative capacity, avoiding AICD, and promotes T cell reactivation on antigen contact after passing to resting state. By this, even a few cells trafficking to tumor could activate and expand in situ to large numbers until tumor elimination, in the same way that virus-specific T cells respond to viral infections. Further, the added costimulation renders designer T cells resistant to regulatory T cell suppression [14].

The benefits of these modifications for improving therapy were enticing, and to many their combination appeared irresistible. With engraftment of 2-signal designer T cells, there would be huge numbers of effectors, and they would never lose their capacity to respond against the tumor threat - or against normal tissues, thereby motivating this essay.

With two independent approaches, however, it is not just their combination but a 2 × 2 array of four distinct Strategies that confronts the investigator in choosing safely how to treat his first patients with a new designer T cell agent: 1^st ^generation or 2^nd^? Infuse or engraft? The philosophy of patient exposures during new drug testing is aimed at proceeding from low risk to higher risk in a regulated fashion. To order these Strategies for risk, therefore, it is instructive to perform a "What-if?" analysis to consider the consequences if G250 designer T cells [2] had had their initial patient exposures under one of these more advanced Strategies.

### "What if...?"

"What if" G250 designer T cells were first applied via ...?

**Strategy 1**. 1^st ^generation, infused [Actual]

In the least aggressive Strategy, infusion of 1^st ^generation G250 designer T cells was seen to mediate significant toxicity. Steroids successfully suppressed the T cell reaction without reactivation after steroid withdrawal.

**Strategy 2**. 1^st ^generation, engrafted

If the same T cells had been engrafted, their resulting vast numbers would likely induce a more severe and possibly lethal toxicity if left unchecked. However, intervention with steroids would again suppress the auto-immune attack. Once brought to resting state and steroids removed, these Signal 1-only designer T cells would be inert (anergic) on contact with antigen positive tissues, and the patient safe from resurgence of his symptoms. Toxicity under this Strategy should be manageable. (See endnote 1.)

**Strategy 3**. 2^nd ^generation, infused

If G250 designer T cells were infused as before but in 2^nd ^generation format, they also would induce toxicity and then respond to steroids. But with removal of steroids, these now-resting 2-signal designer T cells can reactivate on antigen contact with renewed toxicity. Importantly, at low initial exposures in the dose escalation, these infused designer T cells begin as a tiny fraction of the body's T cell repertoire and undergo rapid systemic decline (e.g., 10^9^ cells infused vs 10^12^ total T cells, or 0.1% at peak and lower thereafter). From the analogous clinical setting of donor lymphocyte infusion (DLI), we know that size (of dose) matters, and even with a fully competent allo-immune reaction, small numbers of allo-reactive T cells *can be *safely managed with a balance of GvH reaction and anti-tumor benefit [15,16]. Thus, toxicity under this Strategy should also be manageable.

**Strategy 4**. 2^nd ^generation, engrafted

If 2^nd ^generation T cells had instead been engrafted, G250-specific T cells would not only be capable of reactivation after steroids, but they would be vast in number. With up to 10% of the reconstituted T cell pool being antigen specific after the lowest injected dose (e.g., 10^11^ cells expanding from 10^9^ injected) [17], these cells would be virtually impossible to control, like too high a dose in DLI settings. Maximal immune suppression would be required at all times, with infectious complications and a predictably fatal outcome. Had the initial patient exposure of G250 T cells been by Strategy 4, the consequences could have been dire.

### Strategy Escalation

With these options, it can be seen that there are now choices, not just of dose levels as in typical Phase I drug studies, but of Strategies, with distinct consequences to each. With these Strategies available, how does one best advance the therapeutic aims while remaining faithful to principles of patient protection via an incremental exposure to risk? This brings us to the concept of Strategy Escalation. Strategy 1, simple infusion of 1^st ^generation, is the most conservative; Strategies 2 and 3, engraftment *OR* 2^nd ^generation, are intermediate in risk; Strategy 4, engraftment *and* 2^nd ^generation, is the most aggressive. To proceed from the untested state for a new target ("0") to its most potent implementation, one could envision a Strategy Escalation path of 0 → 1 → (2 or 3) → 4.

But do I advocate that escalations for all new agents first pass through a Strategy 1 test, infusion 1^st ^generation (0 → Strategy 1)? *No, I do not*. If the target was previously tested with a Strategy 1, it *does *provide more confidence of the safety or hazard for the more aggressive strategies. The G250 test by Strategy 1 showed it was unsafe as a target, from which one may forego all more advanced Strategies, thereby sparing patients from more serious injury. Ultimately, however, drugs must be tested for safety in a setting that reflects their potential utility. Sufficient evidence exists from diverse trials with infusion of 1^st ^generation designer T cells to infer that none will be therapeutically successful by Strategy 1, and safety in this format becomes of mainly academic interest. If we instead start with a more advanced Strategy, what rationale could be invoked?

Strategy 2 with engraftment of 1^st ^generation showed considerable benefit in the analogous setting of TILs where simple infusions had not yielded high response rates [12]. The promise of Strategy 3 with 2-signals to sustain an antitumor reaction in situ is an hypothesis based on encouraging preclinical data; clinical trials are just now underway. Both of these have a rationale for realistic benefit to patients where Strategy 1 no longer does. If we bypass Strategy 1 for initial human trials, there *is *more risk with first patient exposures via engraftment (0 → Strategy 2 test) *OR* 2^nd ^generation (0 → Strategy 3 test), but there is also a rationale for controlling toxicities should they occur, as discussed above.

**Figure 1:**
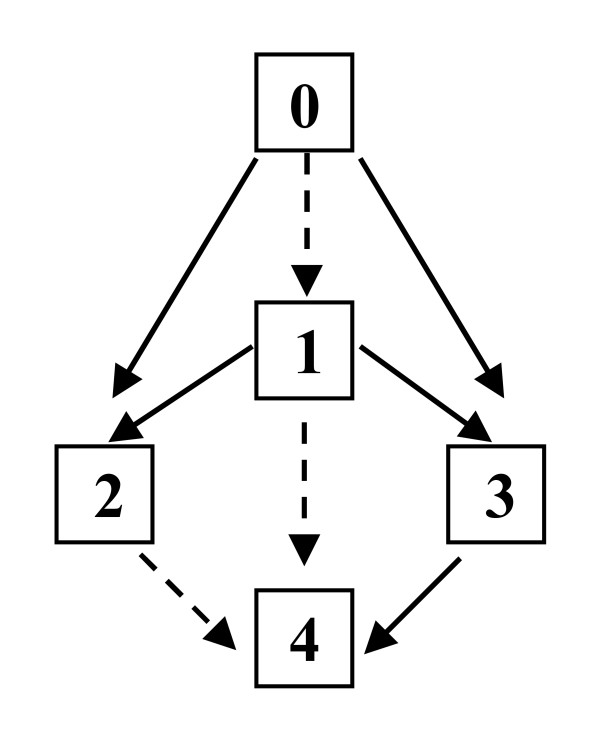
**Safe pathways for Strategy Escalation**. Note that all escalations are permissible except 0 → 4. Dotted paths are proposed as plausibly safe but not advised. See text.

I would argue, however, that proceeding with an untested target (e.g., as was G250) to the most aggressive Strategy 4 (engraftment *AND* 2^nd ^generation) is too much risk. A 0 → Strategy 4 test presumes much about the quality of our knowledge of the potential normal tissue targets and their susceptibility, and, of all Strategies, this one alone allows no exit strategy if we guess wrong. (See Appendix 1 for examples.) No one could foresee the hepatotoxicity of G250 designer T cells [2] or the cardiotoxicity of trastuzumab antibody (Herceptin^®^) [18] prior to the actual human trials. The graded exposures of their respective Phase I/II studies were essential to revealing toxicities before a Grade V event (death). After a target is shown to be safe by one Strategy, one may proceed with fair confidence to more aggressive Strategies, as shown in Figure 1.

### More than safety

Although safer development drives the Strategy Escalation concept, the discipline of this structure can assist in finding more *optimal *development paths as well. For example, while a case can be made for safely escalating T cells from a prior Strategy 1 or 2 to Strategy 4, these paths are not necessarily recommended (dotted in Figure 1). Three reasons *unrelated to T cell safety *may be considered for all paths instead passing through a full Strategy 3 test first:

(1) Lower hazard: The NMA conditioning of Strategy 4 is routinely accompanied by infectious complications that can occasionally be fatal [[19,12]; see also Appendix 1: Designer T cell study deaths];

(2) Lower cost: The clinical (non-manufacturing) costs in the real-world hospital setting are in the range of $4-$8,000 for simple infusion (Strategy 3) versus $60-$100,000 for engraftment protocols (Strategy 4), per our own experience [20-22]; and finally and importantly,

(3) Better science: A direct 0 → Strategy 4 test with engraftment obscures any chance to test the core driving hypothesis of current research, e.g., that additional signals, as embodied in the advanced generation designer T cells, can promote a fully competent T cell response with in situ expansion until tumor elimination.

To this latter point, T cells do this quite efficiently in virus infections without conditioning, and when we have proven ourselves capable to bypass immunization and antigen-presenting cells via this technology, I expect we will prevail similarly with designer T cells against tumor. At the moment that we succeed with the right CARs, such engraftment strategies, with their attendant costs and hazard, will predictably be retired. Hence, in my opinion, engraftment should be viewed as an intervening measure, applied only until we get better at immunology, to compensate for our still-imperfect T cell engineering.

Further, when targeting a normal self antigen, a Strategy 3 infusion may allow "tuning" of the activity against tumor versus normal tissue by judicious dose exposures and a gradation of suppressive therapies (as needed) in the manner of DLI [15], where a Strategy 4 engraftment with its hard-to-control cell numbers may fail. That is, with each new product tested under Strategy 3, an appropriate dose escalation plan affords the best chance to define an optimal biologic dose (OBD) to establish proof-of-concept anti-tumor activity and conditions of safety to normal tissues.

At this point in time, however, the first studies with 2^nd ^generation designer T cells under Strategy 3 (infused) are just coming on-line, and none has yet completed a full escalation with appropriate cytokine support (e.g., IL2). Thus, it is too early to infer sufficiency *or *deficiency of any of the existing 2^nd ^generation reagents to eliminate tumors - without engraftment. But where these more advanced reagents *are *proven therapeutically inadequate (and safe) under Strategy 3 infusions, then engraftment via Strategy 4 with its higher cost and hazard is a justifiable next step in the Strategy Escalation.

**Figure 2:**
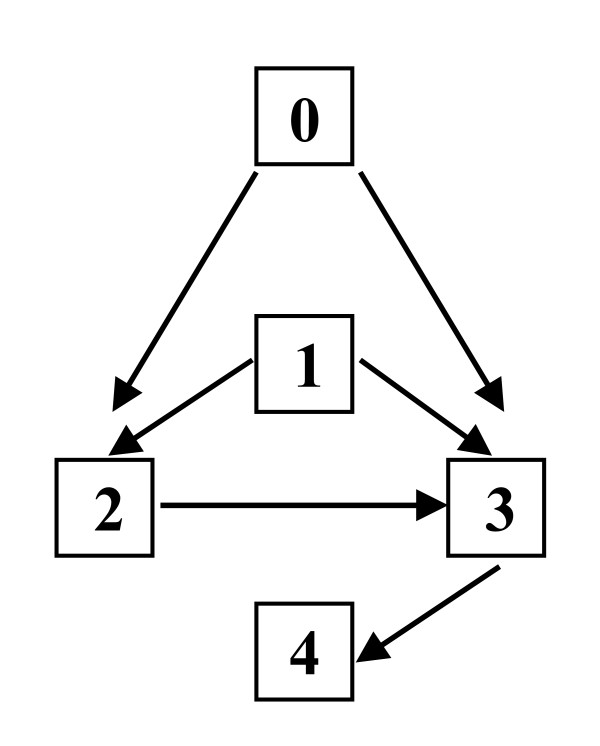
**Optimal pathways for Strategy Escalation**. All paths to 2^nd ^generation engrafted ("4") pass through a full prior test of 2^nd ^generation infused ("3"). See text.

Hence, for untested targets, it is my opinion that Strategy Escalations of 0 → 2 (1^st ^generation, engrafted) or 0 → 3 (2^nd ^generation, infused) are safe and acceptable for initial human exposures. For all targets, tested and untested, I believe for reasons of safety, science and cost that 2^nd ^generation engrafted should instead have a full prior test of 2^nd ^generation infused, i.e., a Strategy Escalation of (0 or 1 or 2) → 3 → 4. (See endnote 2.) This is represented in Figure 2.

## Conclusions

It is recommended that every new immuno-gene therapy proposal be accompanied by a Strategy Escalation discussion that accounts for the molecular and biological features of the modified cells and the method of their proposed administration. This Commentary presents an example of such a discussion from the current state of the art for designer T cell therapies, counseling against the most intensive Strategies for untested antigen targets. If by an early Strategy, the patient can safely be treated, then one may reasonably advance to more potent Strategies with a rationale for safety. Further, it is clear that safety with an antibody is not the same as safety with a T cell; antibody studies therefore cannot substitute for directed designer T cell trials via a less than fully committed patient exposure. As a paradigm, Strategy Escalation is intended to be flexible and adaptive as new therapeutic opportunities are brought forward, e.g., anti-apoptotic genes, suicide genes, co-expressed cytokines, etc., as elaborated in Appendix 2: Future directions. Finally, the formalism of the Strategy Escalation discussion may ultimately find wider application, extending to other cellular therapies as their respective fields mature, e.g., as in stem cells where emerging concerns over options for their safe and incremental application were recently and cogently expressed [23].

## Appendix 1: Designer T cell study deaths

In the past year, two patients died on Phase I designer T cell studies: one targeting CD19 in lymphoma [24,25] and the other targeting Her2/neu in breast cancer [26,27]. Both were previously untested targets for designer T cells. The patients in each case were treated with 2nd generation designer T cells incorporating costimulation, and the two deaths were the first patient in each case to undergo engraftment (Strategy 4). In the former, there was an initial exposure to designer T cells by infusion (Strategy 3) but only to low doses (~10^9^ T cells) without toxicity, and then a death with the first patient to have engraftment of the same dose (0 → (3) → 4 test). (3 in parentheses because it was not a full dose-escalation test.) Was this death due to on-target toxicity (i.e., against CD19 on undefined normal tissue)? In that case, was the jump too big from 10^9^ cells infused on Strategy 3 *transiently *present to 10^11^*stably *engrafted on Strategy 4 (from 10^9^ cells dose)? (See endnote 2.) Or was this death unrelated to any on-target toxicity, perhaps secondary to the conditioning? These questions could not be definitively answered. The study was ultimately allowed to proceed with the second patient treated at half-log lower dose without toxicity [24].

In the second case, targeting Her2/neu, the first patient exposure was a moderately high dose of 10^10^ designer T cells infused after conditioning. This was the first-in-human designer T cell test against this target (0 → Strategy 4 test). The patient experienced acute pulmonary edema within the first hour post infusion, and high dose steroids were initiated. The patient died after five days with cardiac arrest and hemorrhagic enteritis, the latter a recognized manifestation of severe GvHD. Her2/neu is known to be expressed on lung and bowel [28], and may be inferred at low levels in heart by the cardiotoxicity seen in a minority of patients treated with trastuzumab (Herceptin) [18]. This study is presently suspended.

One may consider whether these are second and third examples of antibody therapy being relatively safe (i.e., anti-CD19 antibody [29] and trastuzumab [30]) but designer T cell therapy against the same target is toxic. From the details presented, the likelihood is the CD19 death was not due to T cell toxicity, but rather a complication of the conditioning regimen, a reminder that conditioning, integral to Strategies 2 and 4, is not a benign option. On the face of it, the Her2 death appears to be on-target toxicity in normal tissues, similar to the G250 study [2], but not reversible by steroids due to vast self-reactive T cell numbers in the Strategy 4 setting. An alternative in each case would have been to start with a full Strategy 3, escalating until 10^11^ cells infused, if tolerated, and then switch to Strategy 4, engrafting - but only if Strategy 3 is ineffective. In both instances, these deaths alert us to the potential for serious impact of our interventions, and that the choice of how we incrementally expose patients (i.e., Strategy) may be important to patient safety in a new therapy.

## Appendix 2: Future directions

One may consider the structure of the 2 × 2 matrix for Strategy Escalation as deriving from inherent elements of T cell biology. One dimension is how many T cells there are ("quantity", e.g., Strategy 1 → 2; T cells increased by engraftment) and the other dimension is how effective/potent they are ("quality", e.g., Strategy 1 → 3; T cells more effective with costimulation). This matrix works well for the current state of the art represented in current clinical trials, but new permutations in these strategies are continually being invented. It is instructive to consider how these newer configurations may affect the application of this matrix.

The matter of when to assign a new intervention a new Strategy number (e.g., 5) comes down to *whether an earlier trial needs to be performed before escalating to the new Strategy: *e.g., to address safety concerns of a modification or to serve better hypothesis testing. In most instances, however, it can be seen that these anticipated modifications are still covered under one of these four basic Strategies. That is, novel interventions may be conceptualized along these same two axes of number (quantity) and/or potency (quality), without dramatic changes in the risk implications for untested antigens. These can be annotated with + or - on a basic Strategy number (e.g., Strategy 1+ or 4-) when safety features are considered not to mandate a separate trial. Ultimately, whether a configuration is a Strategy 4+ or a Strategy 5 (needing a Strategy 4 trial first) can be a judgment call for the investigator, but the formalism of the Strategy Escalation discussion provides an explicit framework in which to support that assignment. *In the end, however, the way the Strategies are numbered is less important than the structure that encourages their formal consideration as a strategy*.

In the following, we consider several Strategy configurations that have been described in preclinical work that may find their way into the clinic.

### Multiple co-stimulatory molecules

CD28, 4-1BB, OX40 and others. I have defined all of these constructs, single or multiple, as 2^nd ^generation: they all make T cells more potent (quality), some more than others. The best co-stimulation combinations will make T cells quantitatively more able to mediate toxicity, possibly at lower starting cell exposures, but do not introduce qualitatively novel risks. Unrecognized toxicities against self-tissues should still be adequately covered via infusions (Strategy 3) under a dose-escalation plan with appropriately low starting doses, as in tuning donor lymphocyte infusions (DLI) [15]. Similarly, risks with engraftment (Strategy 4) are not qualitatively different among different 2^nd ^gen constructs once proven safe in a Strategy 3 test.

### Co-expressed cytokines

This falls into two categories: ***Growth factors ***(e.g., IL2, IL7, IL15) and Immune Modulators (e.g., IL12, IFNg). Growth factors constitutively expressed improve cell numbers (quantity) by prolonging T cell survival/expansion. Critically, none has been associated with T cell immortalization. For infusion protocols, the impact on quantity is incremental and manageable (versus the quantum changes for engraftment) and likely does not create new types of risks for 1^st ^or 2^nd ^generation when infused. (See endnote 3.) ***Immune modulators ***like IL12 make T cells more potent (quality) without affecting cell numbers. The anti-self potency can be managed by the same dose escalation as DLI protocols (above). By this Strategy discussion, it appears that there is no untoward risk by Strategy 1 or 3 infusions. Where these cytokines take on special significance, however, is in engraftment protocols. With 10^11^ or more cells post-recovery secreting cytokine, high systemic exposures may create a risk that is off-target and potentially life-long. With this qualitatively new risk, such a study might merit designation as a Strategy 5 protocol, to be conducted post Strategy 4, if ineffective. (However, see below, *On-Off gene control*.)

### Reactivation modulators

Antigen-Fc molecules have been shown to stimulate designer T cells, 1^st ^or 2^nd ^generation, in the presence of monocytes that crosslink Ag-Fc and supply B7 for CD28 engagement and costimulation [31]. This molecule may in principle be used in vivo to reactivate and expand designer T cells in conjunction with any Strategy (1 and 3, post-infusion; 2 and 4, post-engraftment). The ability to control the dose and duration of Ag-Fc exposure allows assignment of Strategy 1+ or 4+, for example, without major risk increment.

### Anti-apoptosis genes

Anti-apoptotic genes can replace growth factors (e.g., IL2) by blocking apoptosis from cytokine withdrawal, e.g., via Bcl-xl over-expression [[32]; Emtage & Junghans, unpublished data], impacting therapy along the cell number axis (quantity). This has the advantage of avoiding systemic cytokine exposures, whether exogenous or expressed in the T cells (above). However, the potential for transformation and immortalization with a Bcl family member [32] distinguishes this class from the expressed cytokines. This introduces a qualitatively new risk, meriting designation as a Strategy 5 protocol, to be tested (with appropriate rationale) only after failure of a prior Strategy 3 or 4.

### Suicide genes

This measure would be unnecessary for most infusion protocols, where the dose escalation and suppressive measures provide adequate protection as discussed in the main text (an exception might be with anti-apoptosis genes). The fail-safe feature of incorporated suicide genes presents a potential escape from any toxicity, however it manifests [33]. In the most relevant clinical model, herpes TK (hTK) has been employed in allo-transplant, where it has successfully combated serious GvHD [34]. In the case of 2^nd ^generation engraftment, a suicide gene could take a Strategy 4 down to a Strategy 4-. Yet, even here, the investigator will want to consider the rapidity and completeness of the suicide (for hTK, hours to days, depending on T cell cycling) versus the rapidity and intensity of onset of adverse effects. In the Her2 study, with a moderate (10^10^) dose of T cells, the patient had respiratory distress by 15 minutes post-infusion, requiring intubation, and was dead in 5 days. (See Appendix 1: Designer T cell study deaths.) A suicide gene could not have prevented the initial event but perhaps the ensuing death. Thus, the option of suicide gene control of non-hyperacute toxicities could take the designer T cells under Strategy 4 engraftment to a risk level approaching simple infusion (e.g., Strategy 3+) by reducing effector cell numbers (cell numbers being the essential difference between 3 and 4). However, it does nothing to improve safety or expense of conditioning, or to correct a muddled hypothesis test with the combined approach. The suicide gene ablation for serious toxicity in engraftment also loses the opportunity to "tune" the therapy in the manner of DLI, available to infusion protocols (e.g., Strategy 3), where a balance of anti-self and anti-tumor activity may be achieved with patient benefit [15]. Lastly, if fully tested under Strategy 3, where suicide genes are generally unneeded, a 2^nd ^generation designer T cell does not require a suicide gene in a subsequent Strategy 4 because safety of the target was previously established.

### On-Off gene control

In analogy to suicide genes, parallel descriptions could be made for control of genes desirable for expression (e.g., of cytokine) that is time-limited without terminating the T cells, allowing for resumption of activity at a later time if needed. Thus, an engrafted 2^nd ^gen designer T cell with co-expressed cytokine under a Tet-On promoter [35], potentially termed Strategy 5 because of the added risk of systemic cytokine, is downgraded to a Strategy 4+ because of the potential to shut off growth factor on Tet withdrawal, thereby avoiding need for a prior Strategy 4 trial for patient safety.

## Endnotes

1. This inference of toxicity manageability under Strategy 2 is consistent with observations in two non-designer T cell studies. TCR transfer engages CD3 Signal 1 on antigen contact, similar to 1^st ^generation designer T cell CARs. Engraftment of T cells with MART1 specificity in a Strategy 2-like application had on-target toxicity that safely responded to steroids [36]. Engraftment with a CEA specific TCR designer T cells also showed on-target normal tissue toxicity that was safely managed [37]. 1^st ^and 2^nd ^generation TCR-based CARs have been created [[38,39]; AJ Bais & RP Junghans, unpublished data] and will engender the same types of discussion as for the Ig-based CAR constructs.

2. Bearing in mind that there is a 100-fold expansion of T cells for the lowest useful doses in the engraftment protocols (e.g., 10^9^ cells) [11,17], it is likely that a reasonable Strategy Escalation increment to a starting test with 10^9^ T cell *engrafted *is not preceded by a test of 10^9^ T cells infused, but by a test of 10^11^ T cells infused. In the latter case, one is comparing 10^11^ T cells *transiently present *by infusion versus 10^11^ T cells *stably present *by engraftment. By moderate increments in risk, the hope is that toxicities will be revealed at less than Grade V (death) on their first expression. See Appendix 1: Designer T cell study deaths.

3. IL7 and IL15 are transiently elevated post-conditioning and thought to drive the homeostatic expansion and engraftment of T cells [12,40]. One might be concerned that these cytokines constitutively expressed in designer T cells could drive T cell expansion without limit. Against this, however, is the observation that engraftment depends upon an empty compartment that is enumerated for TCR populations, independent of the cytokine response [41]. Prudence would dictate, however, that this inference of safety be tested preclinically in vitro and in vivo prior to human exposures.

## Competing interests

The author declares that he has no competing interests.
